# Contact Logger: Measuring everyday intergroup contact experiences in near-time

**DOI:** 10.3758/s13428-019-01335-w

**Published:** 2020-01-21

**Authors:** Tina F. Keil, Miriam Koschate, Mark Levine

**Affiliations:** 1 Department of Psychology, University of Exeter, Exeter, UK; 2 Department of Psychology, University of Lancaster, Lancaster, UK

**Keywords:** Intergroup contact, Mobile smartphone technology, Usability, Near-time data collection, Everyday contact, Intergenerational contact, Experience sampling, Ecological momentary assessment

## Abstract

Intergroup contact research has traditionally relied on retrospective accounts of intergroup encounters, mainly through survey-based or observational methods. This study introduces and tests the usability of a purpose-built, location-aware mobile application—the Contact Logger. This application enables the recording of interpersonal and intergroup encounters, in public and private spaces (both indoor and outdoor), in their here-and-now contexts. The main advantage of this approach, as compared to traditional methods, lies in its ability to collect repeated and timely (near-time) self-assessments of individuals’ behaviors and experiences. It also allows for geographical location data to be logged. Usability testing was conducted in a real-world environment and took place over the course of seven days, during which participants (*N =* 12) logged every contact they had with an outgroup member (here, older people). Subsequently, participants completed a paper-and-pencil questionnaire, reporting on the usability and experience of using the Contact Logger. The results showed that the application is a viable and easy-to-use alternative to traditional methods. The information gathered aided the further development and optimization of the application. The outcomes of this development process are also briefly discussed.

Intergroup contact is one of the most widely studied and effective interventions for prejudice reduction (Dovidio, Love, Schellhaas, & Hewstone, 2017; Tropp & Page-Gould, 2015). Since Pettigrew and Tropp’s (2006) meta-analysis established that contact quantity is negatively related to prejudice in various intergroup contexts, attention has turned toward better understanding the conditions under which positive intergroup contact can improve intergroup interactions and reduce prejudice. Research has started to look at structural variables, such as institutional support (Koschate & van Dick, 2011) and social structure (Eller, Abrams, & Koschate, 2017), as well as individual-level processes, such as the propensity for individuals to engage in intergroup contact (Hodson, Turner, & Choma, 2017). Furthermore, comparisons from findings from related areas such as the intergroup interactions literature (MacInnis & Page-Gould, 2015) and diversity research (Wessel, 2009) are also attracting research attention (Christ et al., 2014).

This shift in research focus has led to a renewed interest in the direct interactions of individuals in spaces in which intergroup contact takes place (Dixon, Tredoux, & Clack, 2005; Dixon, Tredoux, Durrheim, Finchilescu, & Clack, 2008; Foster, 2005; McKeown & Dixon, 2017). However, as MacInnis and Page-Gould (2015) and Thai and Page-Gould (2017) point out, methodological barriers that make it difficult for researchers to examine the breadth and depth of intergroup contact in real-life situations remain. Whereas much of the intergroup contact literature uses retrospective self-report measures that aggregate across individual contact interactions, intergroup interaction studies use artificial laboratory settings to study exchanges between outgroup strangers. Neither of these approaches studies the dynamics of intergroup contact—that is, how different contact experiences interact to affect prejudice and other relevant outcomes—nor how intergroup contact is situated in physical space.

In the following sections, we will briefly discuss current approaches to measuring intergroup contact and their limitations for present intergroup contact research. We will then introduce a new methodology of near-time self-reports of outgroup contacts using a mobile phone application. We report a usability study of this method, showing that it is a viable alternative to paper-and-pencil methods that offers new ways of examining intergroup contact and intergroup interactions.

## Retrospective versus near-time methodologies

As Pettigrew and Tropp’s (2006) meta-analysis of over 500 intergroup contact studies revealed, most intergroup contact research makes use of self-reports^1^ (81%, according to Hewstone, Judd, & Sharp, 2010). Easily administered and relatively inexpensive to carry out, self-report methods provide a valuable tool to access respondents’ inner states (Christ & Wagner, 2012). However, the method has been criticized for several important limitations (e.g., recall bias; social desirability, acquiescent and extreme responding). Despite the well-documented limitations of self-reports, the validity of intergroup contact studies that rely on self-reports is seen to be robust against context effects, to be generally in agreement with observer-based reports, and able to reliably predict contact (Sharp, 2013; Sharp & Hewstone, 2010).

In addition to the reliance on self-report methodologies, the majority of intergroup contact research (70%^2^ up to 2006, according to Pettigrew, 2008) also relies on retrospective accounts. The gap between the event and actual data recording means that information on the immediate experience and situational context of individual contact remains somewhat limited. Given that the majority of past intergroup contact research has focused on direct, face-to-face contact (Pettigrew & Tropp, 2006) with more or less well-known outgroup members, problems related to recall bias may not be particularly severe. By focusing on the aggregate-level validity and reliability of retrospective data, recall bias can be overcome if the sample is representative enough (Reuband, 1994). However, the ability to recall encounters of a fleeting nature, such as interactions with strangers in public settings, presents new challenges. Intergroup encounters in public settings are abundant and often involve contact with unfamiliar outgroup members (e.g., contact with a shop assistant). Unless related to a particularly unusual or meaningful experience, accurate recall of such interactions is likely to be low (see Castel, Nazarian, & Blake, 2015; Fiske, 1995; Robinson & Clore, 2002; Schwarz, 2007). Furthermore, although the aggregation of data alleviates problems with reliability, it conceals any meaningful variation of experience, in particular, the quality of contact experiences. As the recent literature on positive and negative intergroup contact has shown (Barlow et al., 2012; Graf, Paolini, & Rubin, 2014), asking participants to report experiences in an aggregate manner hides important insights into the contact–prejudice relationship that are vital for contact as a successful intervention.

Here, the ability to measure contact in near time and in situ promises to remedy some of the limitations mentioned above. *Near time* refers to the capturing of information directly after an event, rather than while the event is happening. Often, self-reporting during an event or interaction would be disruptive and might be perceived as inappropriate by an interaction partner. In contrast to retrospective reporting, near-time data collection reduces recall bias and facilitates the capturing and analysis of intergroup contact with maximum ecological validity in everyday, real-world contexts (Shiffman, Stone, & Hufford, 2008; Stone, 2007). It also facilitates a more fine-grained analysis of attitude dynamics (Bohner & Dickel, 2011; Brousmiche, Kant, Sabouret, & Prenot-Guinard, 2016).

However, near-time data collection comes with its own unique challenges and problems. For example, participants need to remember to record the intergroup contact experience, as they cannot be prompted to do so. Also, knowing that they need to record the contact may affect their experience and behavior. Whereas the latter problem is inherent to any method that seeks to assess experiences in near time, the former problem can be addressed, to some extent, by modern technology. In many parts of the world, smartphones are now ubiquitous (Newzoo, 2018; Poushter, 2016). For many, they have become an indispensable companion that satisfies informational and recreational needs (Fullwood, Quinn, Kaye, & Redding, 2017). For research, smartphones can be used to automatically record a multitude of information, such as the date, time, duration, and geographic location.

## The Contact Logger

In recent years, more and more tools are becoming available that enable the repetitive sampling of individuals’ behaviors and experiences. Open-source variants include, for example, the AWARE Framework (Ferreira, Kostakos, & Dey, 2015), ExperienceSampler (Thai & Page-Gould, 2017) and more recently MobileQ (Meers, Dejonckheere, Kalokerinos, Rummens, & Kuppens, 2019). Such self-reporting tools are examples of ecological momentary assessment (EMA) and experience-sampling methods (ESM) (Larson & Csikszentmihalyi, 1983).^3^ The main advantage of these methods is that they allow researchers to study and address how behavior and (emotional) experiences change over time and across contexts within their natural environments (Shiffman et al., 2008).

As the cost of mobile technology continues to decrease, and the development of mobile applications requires less specialist knowledge, technology-based versions of EMA have become increasingly popular (see Firth, Torous, & Yung, 2016; Heron, Everhart, McHale, & Smyth, 2017, for a systematic review of mobile-technology-based EMAs; and Kuntsche & Labhart, 2013, who outline some of the advantages of this approach). However, ESM mobile apps such as ExperienceSampler (Thai & Page-Gould, 2017) have so far rarely been used in intergroup contact research. Although it is possible, in principle, to use ESM apps like ExperienceSampler for intergroup contact research, they were designed for a different purpose, and hence lack some of the functionality and usability that would make such an app a powerful tool to assess intergroup contact. For instance, ESM apps often need advanced programming skills to customize them and add in functionality specific to intergroup contact research: that is, they are not “ready to use.” For example, the layout of questions and scales in such apps is often generic. Although this makes it easy for researchers to customize the app, they are not optimized for usability within a particular research context, and their survey-like format means that answering can be time-consuming. Moreover, additional features are often provided as third-party plug-ins, which can make maintenance and control of coding quality difficult. Finally, geographical location detection is often not standard. Those that do offer built-in location tracking are often limited to (outdoor) tracking via GPS only and track continuously, rather than recording the precise geographic location of a specific contact event.

Therefore, we decided to develop a purpose-built app as the efficiency, and a minimally intrusive method of logging is essential for participant compliance. Moreover, geographical location data substantially add to the usefulness of the app for intergroup contact research. The Contact Logger uses a mobile technology-based EMA-like assessment to allow researchers to measure and record each and every intergroup contact event, including its indoor or outdoor geographical location, representing a novel method for intergroup contact research.

## Aims and objectives

The overall aim, thus, was to develop a tool that could be used to measure intergroup contact in private as well as public settings, in near time. The first step in the development of such a tool was to define the basic criteria that it needed to fulfill. For the aims of this project, these included the abilities (a) to record the attributes of multiple contact events; (b) to measure contact in a manner that keeps interference with the contact event and the participant’s daily routine to a minimum; (c) to develop a tool that is intuitive, practical, quick, and easy to use; and finally (d) to determine and record the precise geographic contact location, in outdoor and indoor environments.^4^

Unlike typical ESM studies, the app does not prompt participants to log contact at certain preset intervals. Contact in natural settings can occur at any time. Instead, participants are asked to log a contact directly after it naturally occurs in order to record data in near time. Furthermore, our decision to develop a native app was also based on the wish to have full control over the app’s design, performance, and maintainability. In contrast to hybrid or web-based apps, native apps are specific to the phone’s operating system (OS). Hence, separate apps need to be developed for the most common OSs currently available (i.e., for Android and iOS phones).

Thus, the first objective was to create a beta version that could be tested in the field; the second was to learn and apply the knowledge gained from the field test (usability study), which would result in the release of a stable and reliable version, suitable for research in a real-world environment.

## Development considerations, materials, and process

### Hardware

After consideration of other available technology, it was decided to develop a custom, context-aware, event-contingent, experience-sampling application (Wheeler & Reis, 1991), in the form of a smartphone application (app). Android was chosen as the initial development OS, as it offered technical and methodological flexibility at the best cost–performance ratio. The ability to accurately detect geographic locations depends on many factors, such as the environment and the hardware used to receive signals from a global positioning system (GPS). Depending on make and model, mobile phones are equipped with different GPS chipsets, which can affect GPS sensitivity and accuracy (von Watzdorf & Michahelles, 2010). To enable an evaluation of the app’s location detection accuracy and related battery-usage, we decided to equip all participants with the same devices. However, further testing should be carried out across a variety of different hardware and OS versions, so that future versions of the Contact Logger can be used with participants’ own devices (Haeng-Kon, 2016). Accordingly, Motorola Moto-G Smartphones, with Android 5.1 as the OS, were acquired.

### Programming environment and tools

The app was programmed using an open-source integrated development environment,^5^ which provided all necessary tools to build a scalable native Android application. Initial application development took approximately four months. Bitbucket,^6^ using Git, was used as a software revision control system.

### Application specifications

**Fig. 1:**
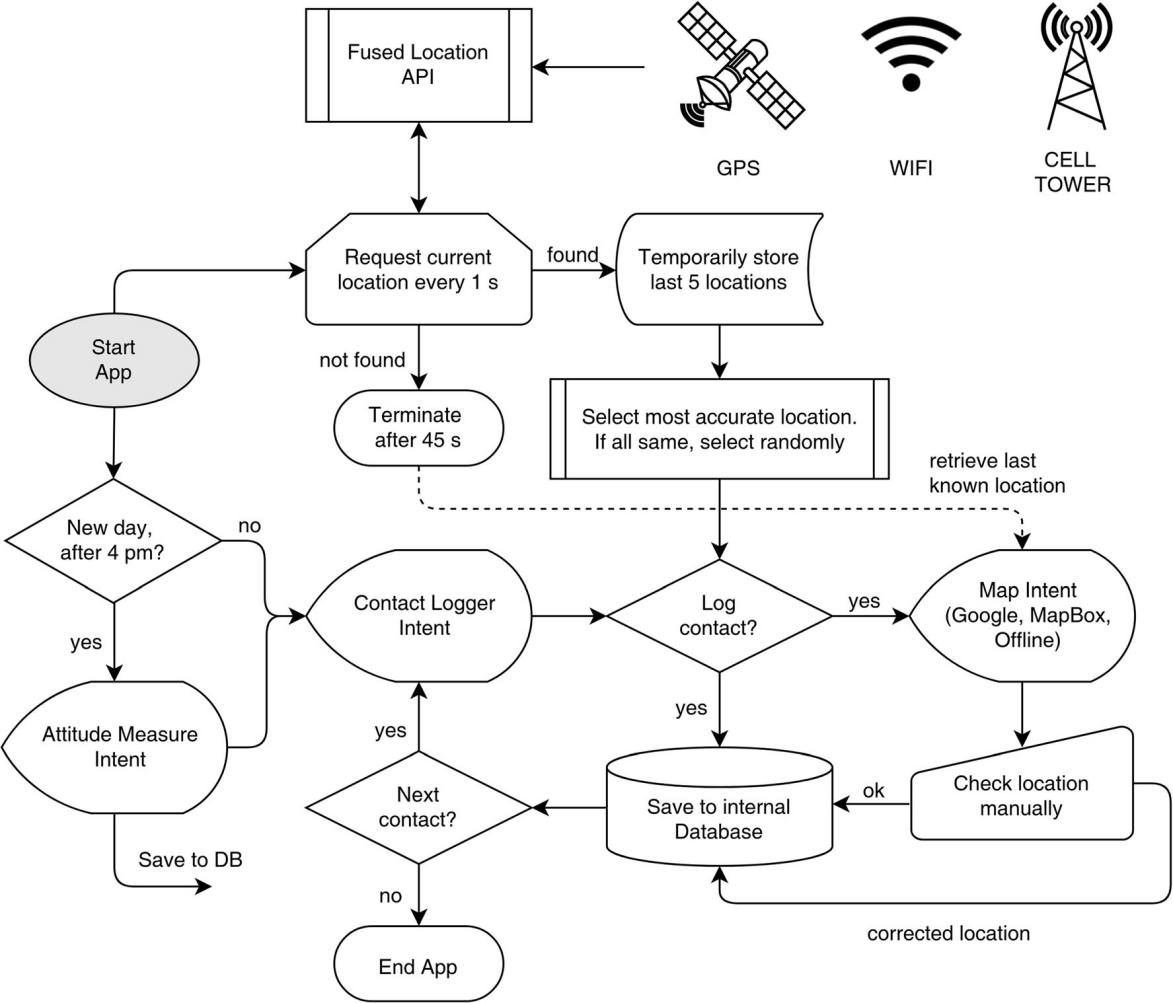
Contact Logger—Application process logic.

An essential requirement of the app was that it should be able to determine a participant’s location as quickly as possible irrespective of the environment. However, determining a location solely via the phone’s built-in GPS receiver is problematic as signal quality is only strong enough outdoors. Further, continuous GPS use can quickly drain the phone’s battery. Fortunately, locations can also be determined via cellular and wi-fi signals, making indoor detection possible. However, the location accuracy determined via cell-tower and wi-fi networks varies widely, ranging from two to three meters to several kilometres (Giaglis, Kourouthanassis, & Tsamakos, 2003; Zandbergen, 2009). Therefore, it was decided to use Google’s Fused Location Provider application programming interface (API), which optimizes power consumption, improves accuracy, and expands coverage by bringing together cellular, wi-fi, and GPS location data. To ensure that the location was determined as quickly as possible, the detection process and algorithm began as soon as the app was started (see Fig. 1). On average, it took approximately 15–20 s to log a contact,^7^ thus giving the detection process plenty of time to determine the current location. In certain circumstances, however, the app may still fail to detect a participant’s location accurately. Participants were thus shown the identified location after logging contact, with the option of correcting the location.

Additionally, a variety of map layer styles from different online map providers were selectable (e.g., Google, Mapbox, Openstreet). In the rare event that online access was not available, a locally installed offline map was shown.

**Fig. 2:**
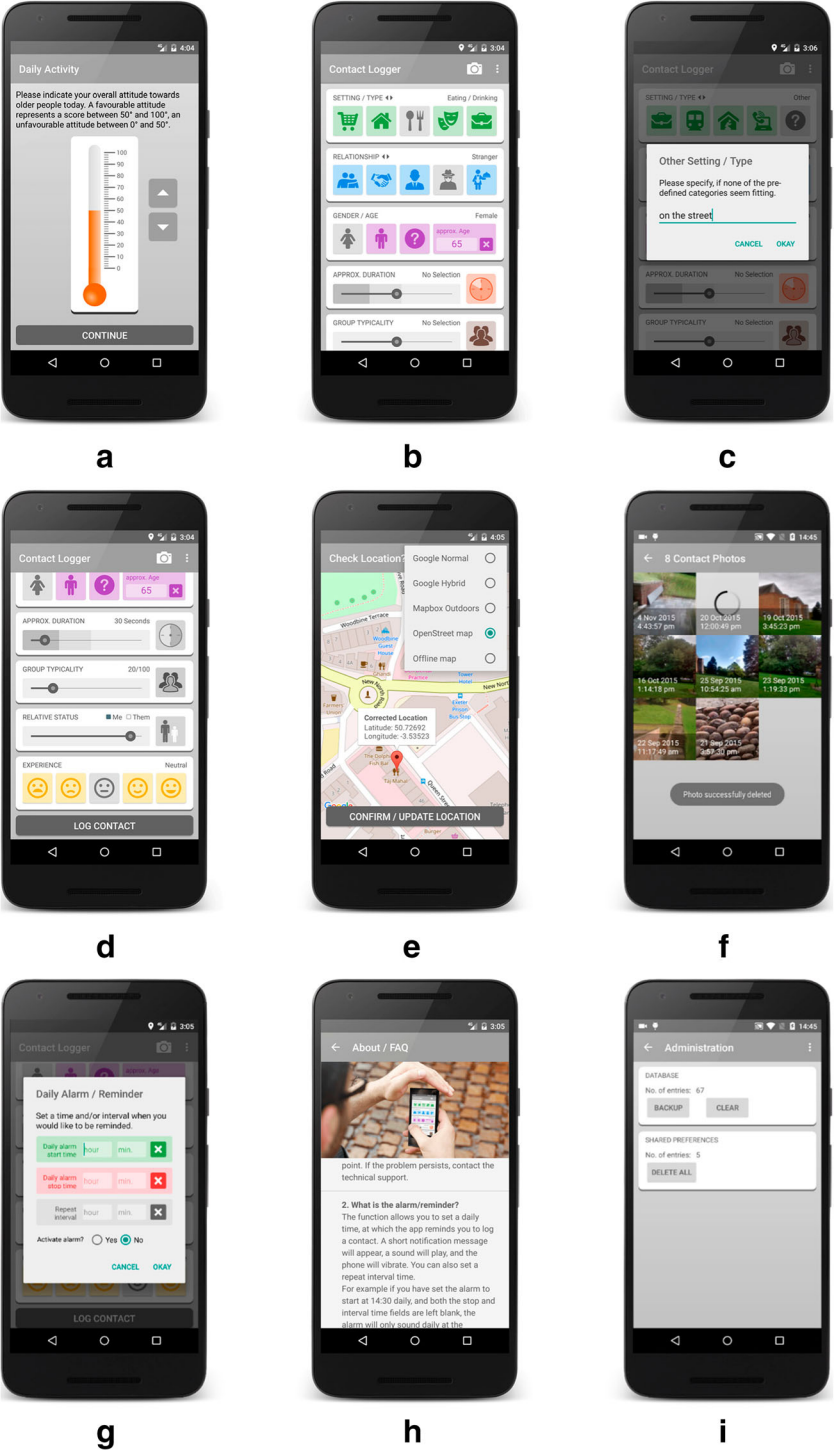
Screenshots of the Contact Logger application (Version 1): (a) daily activity, shown once a day, after 4:00 p.m.; (b) contact details; (c) custom contact type; (d) contact details continued, scroll view; (e) current location, with map type selection; (f) contact photo option (optional), with overview of photos taken, including the delete option; (g) daily alarm/reminder (optional); (h) help section (FAQ); and (i) password-protected administration area.

The collected data were stored in an SQLite database on the phone’s internal SD card. A password-protected administration backend provided functions to export (back up), delete, and reset the stored data (see Fig. 2). Local data storage avoids the need for an external online storage solution, thus reducing privacy and security concerns.

## Usability testing study

The purposes of the usability study were to test the app’s technical functionality and the suitability of the measures and scales employed and, most importantly, to ascertain the participants’ overall experience of using the app on a daily basis (Flood, Harrison, Iacob, & Duce, 2012; Nayebi, Desharnais, & Abran, 2012). This is in line with the definition of usability introduced by the International Organization for Standardization (2010). Usability testing of mobile applications is often carried out in a laboratory environment. While this is advantageous, in that the user’s interaction with the device can be closely monitored (e.g., through an observer or a video camera) and guided by assigning participants specific tasks, a laboratory setting often does not reflect, nor produce, the kinds of problems that may be encountered in real-world contexts (Baravalle & Lanfranchi, 2003).

The primary focus of this study was therefore not on the statistical analysis of the contact data in relation to intergroup contact theory, but on the suitability of the app as an alternative and new method for collecting contact data in situ.

### Method

The optimal sample size for usability studies varies considerably and depends on the chosen methodology and overall aims of the study. In usability-testing research, a sample size of 10–12 participants is considered a reasonable baseline range for studies that mainly focus on problem discovery (Macefield, 2009). Typically, ten participants are enough to detect between 82% and 94% of all usability-related problems (Faulkner, 2003).

#### Participants

A total of 12 participants (75% female) between 21 and 50 years of age (*M* = 30.67, *SD* = 7.56) took part in the usability study. The sample included students and staff from the University of Exeter, and one family member of a student, with a variety of different ethnic backgrounds (Europe, East Asia, and Middle East). Participation was entirely voluntary, and remuneration was neither offered nor given.

#### Materials and procedure

All participants provided written informed consent, and all data were held in accordance with the principles of the Data Protection Act of 1998. After consent, each participant was provided with a smartphone on which the Contact Logger app (Version 1, Fig. 2) was preinstalled and configured. The mobile phone was equipped with a SIM card topped up with 500 MB of online data. To ensure optimal internet access (although the app also works offline), participants were asked to sign into wi-fi access points whenever possible. Before the start of the study, participants were given a link^8^ to a short online video of the app’s basic functions. For the duration of the study, participants were asked to log every encounter they perceived as “contact” with an older person (outgroup member) for a period of one week. An “older person” was described as someone whom the participant believed to be at or near retirement age. Participants were requested to log each contact directly after it had taken place as close to the original vicinity of the contact location as possible. Upon return of the mobile phone, participants were asked to fill out a paper-and-pencil posttest questionnaire, which inquired about the experience and practicality of using the mobile phone and app.

#### Measures: Contact Logger app

In general, the measures chosen for the app were based on typical measures used in traditional intergroup contact research. To keep the logging process as short as possible, single-item measures were used.

##### Attitude toward outgroup

Attitude was measured using a single-item feeling thermometer, ranging from 0 °C to 100 °C (Campbell, 1971; Haddock, Zanna, & Esses, 1993). The measure was shown on a new separate intent (screen) once a day, at or after 4:00 p.m., and was adjustable in steps of one degree by selecting the up or down arrow beside the thermometer (see Fig. 2a).

##### Type of contact

Two separate sets of toggle buttons (on/off), each embedded in a horizontally scrollable frame, allowed participants to indicate the type of contact they had just encountered. Upon selection of a respective button, the color of the button changed to gray, and the text in the right-hand corner changed so as to indicate the selection (see Fig. 2b). The first row of buttons (green icons) conveyed where the contact had taken place and represented the situational context of the contact (i.e., contact while shopping, at home,^9^ while eating/drinking, at a leisure location, at the workplace, while traveling, at a place of worship, while online or on the phone, or other). The second row of buttons (blue icons) indicated the relationship with the outgroup member (i.e., contact with a friend, acquaintance, colleague, stranger, service clerk, neighbor, partner/spouse, relative, or other). By selecting the question mark button (termed “other”), participants were able to enter custom text that described the situational or relationship context on the contact in their own words (see Fig. 2c).

##### Gender and age

The gender of an outgroup member was recorded via the selection of one of three toggle buttons (female, male, other: see Fig. 2b). The approximate age of an outgroup member was assessed using a restricted text field that only allowed the input of a number between 1 and 99.

##### Contact duration

A 24-step horizontal seek-bar widget allowed participants to record how long a contact had lasted. The duration was set by sliding the thumb element from its default position to the required time (min = 5 s, max = 12 h). As an additional indicator, the clock face to the right of the seek-bar reflected the selection. Selection of the clock face reset the seek-bar to the default “no selection” position (see Figs. 2b and 2d).

##### Group typicality

The perceived typicality of an outgroup member was assessed using an 11-point horizontal seek-bar widget. The intensity of perceived group typicality was indicated by sliding the thumb element from its default position toward *not at all typical* (0) or *very typical* (10). With increasing typicality, the person in the right-hand icon became darker, blending in more and more with the background group. Selection of the icon reset the seek-bar to the default “no selection” position (see Figs. 2b and 2d).

##### Relative/equal status

Outgroup member status was indicated relative to the participant’s own perceived status. An 11-point horizontal seek-bar, ranging from *much lower status* (− 5) via *equal status* (0) to *much higher status* (+ 5), allowed participants to report this difference. Sliding the round thumb element to the left from its default position indicated that the participant’s status was lower than that of the outgroup member. Conversely, sliding the thumb element to the right indicated that one’s own status was higher than that of the outgroup member. As an additional indicator, the relative heights of the two persons depicted on the icon to the right of the seek-bar reflected this relationship (see Fig. 2d). Selection of the icon reset the seek-bar to the default “no selection” position. To produce a measure of equal status, values toward the outer ends of the scale (lower status/higher status) were recoded as 0 = *unequal status* (irrespective of direction), with equal status being recoded as 5 = *equal status*.

##### Contact quality

Quality of contact (experience) was measured using five toggle buttons depicting a range of negative, neutral, and positive smiley faces (see Fig. 2d). The response scale ranged from *very negative* (1) to *very positive* (5).

##### Geographical location

After a contact event was logged, the participant was automatically shown a map with a red marker that pinpointed the current location (GPS coordinates; see Fig. 2e). The participant then had the option of correcting the automatically detected location by dragging the marker to a different location on the map. Participants who did not wish to reveal their location were informed (in the consent form) not to log the contact. The automatically detected GPS coordinates, the manually corrected coordinates (if applicable), and the determined GPS location accuracy were recorded.

##### Photo (optional)

As part of a project that explored intergroup contact in mixed areas of Belfast, Stevenson and Sagherian-Dickey (2015) successfully asked participants to indicate their use of physical space within their locale by taking photos. Because they suggested that such a function might also be a useful addition for the Contact Logger, it was integrated as an optional app feature. This allowed participants to take a photo of the contact location or anything relating to the contact interaction, without needing to start an external separate camera app. Upon selection of the camera icon at the top of the contact-logging screen (see Figs. 2b and 2d), the mobile phone’s standard camera application opened. Via the app menu (selectable by clicking on the three vertical dots at the top right-hand corner), participants were able to view the photos and to delete them if they wished (see Fig. 2f).

##### Daily alarm reminder (optional)

If required, participants could set a daily alarm that would remind them to use the app (see Fig. 2g). The function could be set to alarm at specified intervals within a restricted time period. The reminder functioned even when the app was closed or when the phone was rebooted. Both vibration and sound notifications were supported.

#### Measures: Posttest questionnaire

##### Information provision

To assess how easy it was for participants to provide information for each app measure, nine items on a five-point Likert scale (1 = *Not at all easy*, 5 = *very easy*) were used. For example, participants were asked how easy it was to provide information about the situational context in which the contact took place (e.g., shop, workplace, etc.), how typical of their group they perceived the contact partner to be, or about the perceived age of the contact partner. Participants were also given the opportunity to provide details about difficulties in providing information on a particular app measure.

##### Logging a contact

Two open-ended, qualitative measures were used to assess how participants felt about logging a contact with the app and whether doing so concerned them in any way. The aim of this measure was to examine whether participants felt anxious, worried or uncomfortable about logging a contact (or particular contacts) with the app. A further open-ended, qualitative measure tapped into the covert nature of logging a contact: That is, whether participants felt comfortable logging a contact when their contact partner was not aware that they were doing so. Participants were also asked whether a contact partner had noticed the logging of a contact and if so, how the participant had responded.

##### Operative and functional usability

To assess how easy it was for participants to use the app, five items on a five-point Likert scale (1 = *Not at all easy*, 5 = *very easy*) were used. The measure enquired about operative issues such as ease of starting/stopping the app, use of photo and location update options, usage frequency, and remembering to use the app. Furthermore, five short, open-ended qualitative items tapped into the more functional aspects of using the phone and app. These included questions regarding battery life, online access, GPS location recognition and accuracy, and use of the app’s FAQ section (see Fig. 2h).

##### Daily usage

To assess possible issues regarding daily use of the app, and the overall implications this may have for participation in a study that uses such an app, two open-ended qualitative items were used to measure whether and how frequently participants had forgotten to take the phone with them, and more generally to assess the experience of using the mobile app and participating in the study.

### Analyses and results

To assess usability, the data from the Contact Logger and the posttest questionnaire were analyzed in a descriptive and qualitative manner, in line with the specified aims of the study. However, the descriptive results were evaluated more in terms of the broad suitability of the app for collecting intergroup contact data, rather than focusing on the analysis of the collected longitudinal and spatial data in terms of what they mean for social psychology related questions. The following sections primarily reflect an evaluation of the app’s usability for research purposes, the suitability of the app’s control elements and measures, and the outcomes of the posttest questionnaire.

#### User interface and control elements

**Fig. 3:**
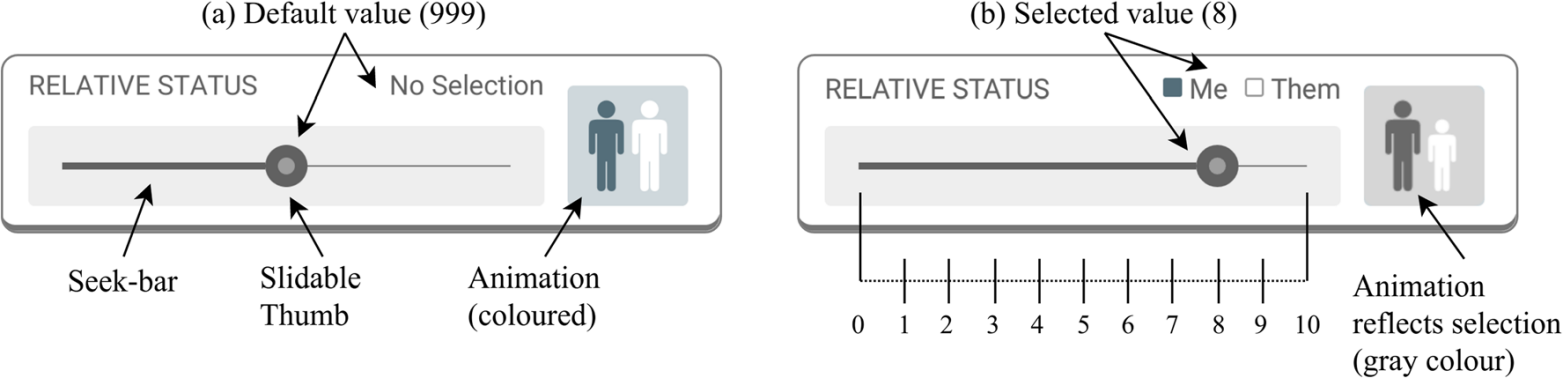
Seek-bar widget components and functionality (app Version 1).

Seek-bar widgets were used to capture contact duration, group typicality, relative/equal status, and attitude (see Figs. 2 and 3). Although controls of this type make optimal use of available screen space and provide an easily configurable segmented or continuous measurement scale, several problems were identified. Firstly, the default setting of the thumb slider was visible at the midpoint of the scale even though the descriptor showed “no selection.” A value of 999 was recorded if the slider was not moved (see Fig. 3). This setting may have unintentionally provided an anchor for participants’ decisions. Furthermore, intentional selection of a midpoint value required participants to drag the thumb either to the left or right and then back to the middle.

Second, due to the stepwise resolution of three of the seek-bars, a smooth and consistent movement of the thumb on these widgets proved difficult. The typicality, status, and duration measures were thus sometimes unresponsive and required several attempts in order to set them to the desired value. Similarly, the red pin marking the participant’s current location on the geographic location map (see Fig. 2e) required the user to long-press on a relatively small icon before it became draggable to a new location. Although a small, removable notice overlaid on the map made participants aware of this functionality, the dragging functionality of the pin was not always an intuitive behavior.

#### Data collection

##### Quantity and type of contact

Of the 107 contacts recorded, 15 contacts (14%) were logged up to three days after the specified seven-day period of the usability study. However, due to the exploratory nature of this study, these contacts were included in the analysis. The highest total number of contacts logged by a participant was *n* = 25; the lowest was *n* = 2. The perceived age of the outgroup members (48% female, 51% male, 1% other) ranged between 55 and 85 years (*M* = 65.3, *SD* = 7.66). The estimated duration of contacts ranged between 5 s and 240 min (*M* = 20.4 min., *SD* = 45.9 min.). A detailed breakdown per situational context and relationship context is given in Table 1.

**Table 1:** Number of contacts per category and context

Situational Context			Relationship Context		
Contact Type	*n*	%	Contact Type	*n*	%
Shopping	5	4.67	Friend	0	0.00
Home	5	4.67	Acquaintance	13	12.15
Eating/drinking	8	7.48	Colleague	24	22.43
Leisure	4	3.74	Stranger	18	16.82
Work	38	35.51	Service clerk	10	9.35
Travel	5	4.67	Neighbor	1	0.93
Place of worship	0	0.00	Partner/spouse	5	4.67
Online/phone	29	27.10	Relative	30	28.04
Other–Public	11	10.28	Other–Exposure	6	5.61
Other–Private	2	1.87	Other–Familiar	0	0.00
Total	107	100.00		107	100.00

*n* = Number of contacts logged.

##### Additional contact attributes and attitude measurement

Table 2 gives a descriptive overview of all other measures logged with the app. The decision to measure attitude on a daily basis in the late afternoon (4:00 p.m.) was based on the presumption that this would give participants enough time to form an overall opinion of their attitude toward the outgroup based on the experience of contacts encountered up to then. However, only 40 (37%) of all contacts took place after 4:00 p.m. This meant that if a participant did not have contact after this time on a particular day, their outgroup attitude for that day was not measured, because the screen with the attitude measure only appeared after this time. Consequently, no participant reported attitude on each and every day of the study. Within this period, outgroup attitude was reported by two participants (17%) on five or more days, by five participants (42%) on two days, by three participants (25%) on one day, and by two participants (17%) not at all. This resulted in a relatively low response rate of 25% (i.e., 23 out of 93 possible responses for 12 participants on seven to ten days, depending on length of phone use).

**Table 2:** Descriptive statistics of outgroup contact evaluations

Measure	*N*	Min.	Max.	*M*	*SD*
Contact quality	106	1	5	3.87	0.79
Relative status	103	− 5	5	− 0.03	1.93
Equal status	103	0	5	3.59	1.32
Outgroup typicality	107	0	10	6.41	2.23
Outgroup attitude	26	50	100	77.27	12.09

*N* = Number of contacts; Results are based on all contacts, including those reported after the seven-day study duration.

##### Location detection

Location accuracy depends on the provider (i.e., GPS, wi-fi, cell tower), and for GPS specifically, on the sensitivity and quality of the hardware (GPS chipset) used to receive GPS signals. The location provider was controlled by the Fused Location API and an additional selection algorithm. An evaluation of the location data revealed an accuracy of *M* = 12.59 m, *SD* = 13.80 m, ranging from 3.89 m to 96 m. However, the accuracy of the information returned by the API is based on a 68% confidence interval.^10^ The detected location was manually corrected 58 times (54% of all logged contacts), by *M* = 4.55 km, *SD* = 32.04 km, ranging from 5.49 m to 244.12 km. A qualitative evaluation of location accuracy is given in the Posttest Questionnaire section.

**Fig. 4:**
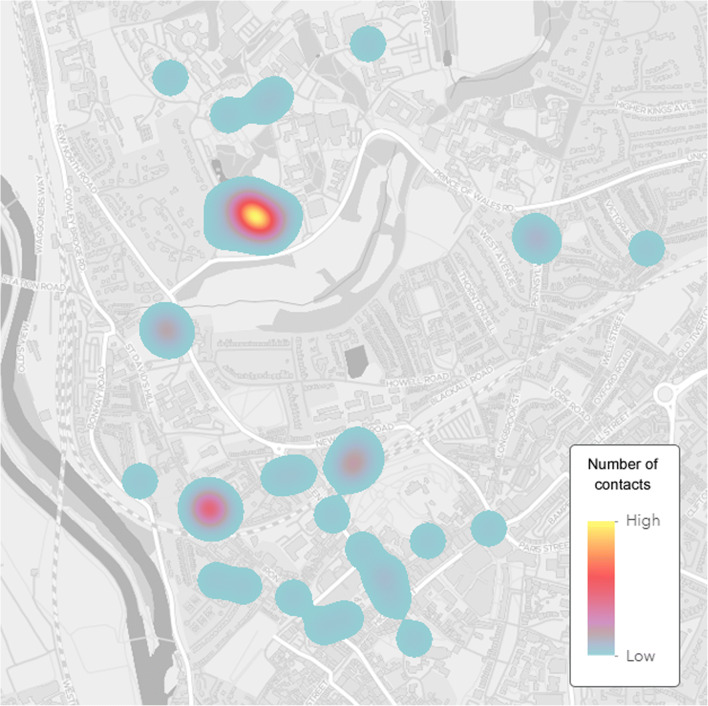
Visualization of outgroup contact density. Basemap source: Esri, HERE, Garmin, ©OpenStreetMap contributors, and the GIS User Community (2011).

**Fig. 5:**
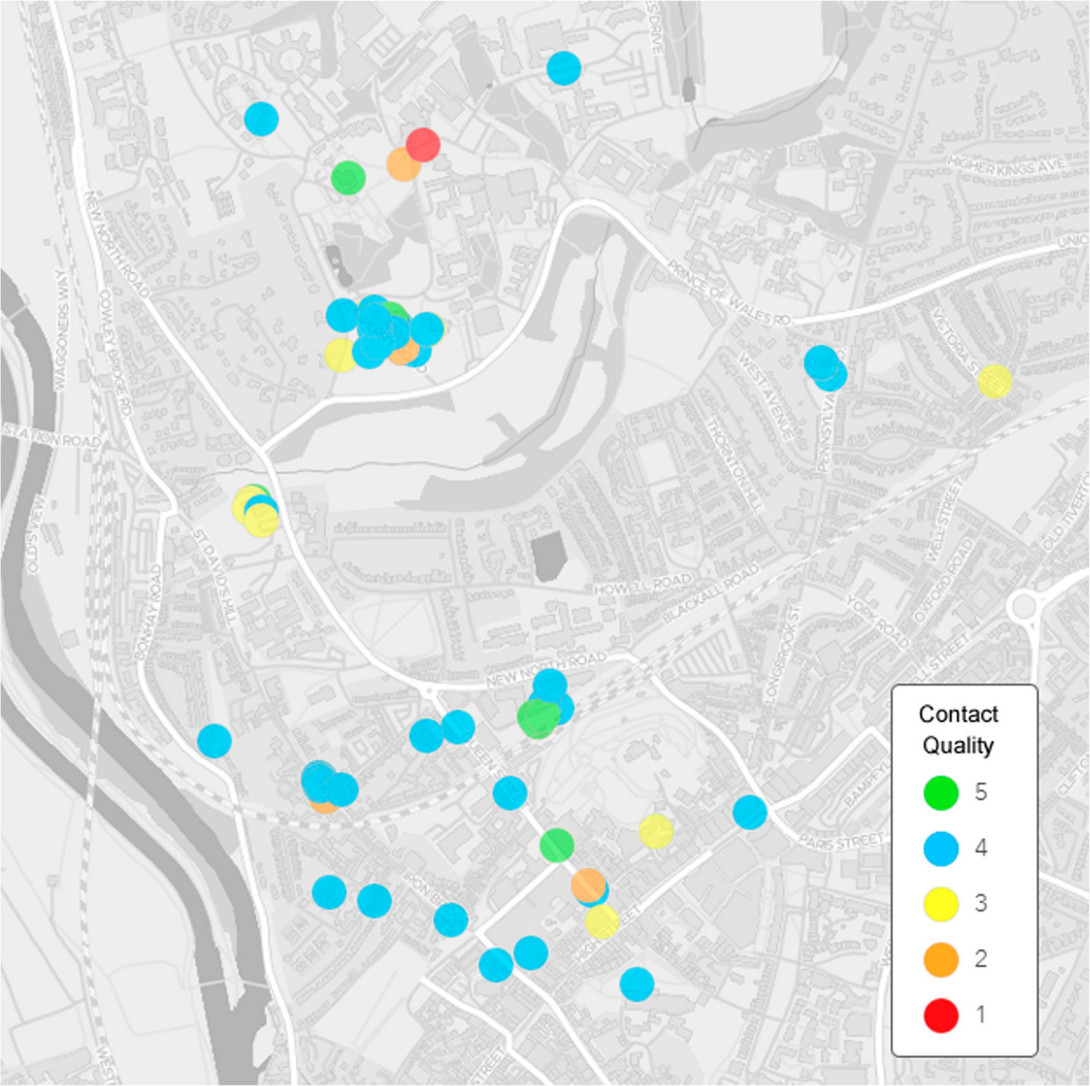
Locatedness of contact quality, from *very negative* (1) to *very positive* (5). Basemap source: Esri, HERE, Garmin, ©OpenStreetMap contributors, and the GIS User Community (2011).

Figures 4 and 5 give an example of the advantages that geographic location data provide. Here, location data (GPS coordinates), in combination with intergroup contact measures, can be used to visualize the relationship between locations and intergroup contact experiences, thus allowing evaluation of the micro-ecology of intergroup contact spaces. For example, Fig. 4 reveals where the highest number of contacts took place, whereas Fig. 5 reveals the locations of positive and negative hotspots of contact experiences.

#### Posttest questionnaire

The overall ratings of the questionnaire’s quantitative measures are summarized in Tables 3 and 4. Qualitative responses were coded using the QDA software^11^ and analyzed using a basic recursive abstraction method (Polkinghorne & Arnold, 2014). Responses were examined for patterns, ordered thematically, and supplement the findings reported in this section.

**Table 3:** Means and standard deviations—Ease of app functions

Measure	*N*	Min.	Max.	*M*	*SD*	*r*
Application start/stop	12	4	5	4.83	0.39	−.157
Daily app usage	12	3	5	4.33	0.89	.137
Remembering to log	12	2	5	3.25	1.14	.235
Ease of taking photos	5	2	5	3.00	1.41	n/a
Ease of correcting location	12	3	5	4.33	0.78	−.257

Scales from 1 = *not at all easy* to 5 = *very easy*; *r* = Pearson correlation with number of contacts per participant.

**Table 4:** Means and standard deviations—Ease of information provision per app measure

Measure	*N*	Min.	Max.	*M*	*SD*	*r*
Contact type/Setting	12	4	5	4.83	0.39	−.194
Contact relationship	12	3	5	4.67	0.65	.195
Outgroup gender	12	5	5	5.00	0.00	n/a
Outgroup age	12	4	5	4.50	0.52	−.322
Approx. duration	12	2	5	3.83	0.94	.075
Outgroup typicality	12	2	5	3.75	1.22	.129
Relative status	12	2	5	3.75	0.87	.367
Contact quality	12	4	5	4.83	0.39	−.194
Attitude	11	2	5	3.91	1.04	.146

Scales from 1 = *not at all easy* to 5 = *very easy*; *r* = Pearson correlation with total number of contacts per participant.

##### Mobile phone handling

A total of 75% of the participants reported having consistent online access. Most of the SIM cards still had over 250 MB of data available at the end of the study, indicating that participants did not use the available online access for private purposes and that the combination of wi-fi access and SIM-card data was more than adequate. On average, participants reported charging the phone twice during the course of the study, whereas six participants (50%) reported charging only once or never. One participant (8%) reported having forgotten the phone on one day because the participant had left it to charge.

##### Forgetting phone

Three participants (25%) reported that they had forgotten the phone on at least one day. The reasons for forgetting included being too tired or too busy (especially at the weekend or after work in the evenings), finding it difficult to remember to use the app after a few days (despite activating the alarm reminder function), and finding it difficult to use a second phone (in addition to their own phone).

##### General app usage

Only two participants (17%) reported having looked at the app’s help section (FAQ; see Fig. 2h). Eleven participants (92%) reported that their location was detected accurately, and only one participant remarked that it was “mostly” shown. Nine participants (75%) reported that the map was always shown after logging a contact. One participant reported that it was “not shown every time”; another that when the online map was not shown, they switched to the offline map; and finally one participant reported that the map was not shown when they were in a location outside of the geographical boundaries of the offline map, which most likely had no online connection.

##### Logging contact

Referring both to the experience and the process of logging contact, eight participants (67%) reported that it was “easy,” “quick,” “convenient,” and/or “fun.” Three participants (25%) noted that after an initial adjustment phase, it became easier and faster to log contact. Nevertheless, one participant (9%) reported finding it difficult to log contacts while traveling, as they needed to concentrate on the modalities of their journey; another that they forgot to log a contact with someone they were too familiar with; and another mentioned forgetting to log phone/email contact altogether. Additionally, although participants were instructed to log each contact directly after it occurred, while still in the vicinity of the contact location, one participant (9%) reported that when they forgot to log a particular contact, they “would do it afterward.” Accordingly, another participant (9%) noted that there should be instructions on whether retrospective logging was acceptable. Furthermore, two participants (17%) reported concerns about logging contact when their parents were involved, noting “My mum would be upset if she knew I considered our [contact] as [an] interaction with an old person,” and “I don’t see him [father] as old, so I hesitated. He started working from a very young age, and even after being officially retired, he is still doing work.” Finally, two participants (17%) felt that “confirmation that logging was successful at the end would have been quite reassuring.”

The process of logging contact made some participants reflect on the amount of contact they had had with the outgroup in general. For example, three participants (25%) reported that they felt that they had little contact with older people, noting that “I didn’t feel I had much contact,” “I realized I don’t have more than average contact,” and “I had very little contact with older people.” Referring to the typicality of outgroup members, one participant (9%) noted that “those I met never felt like category examples.”

##### Control elements

Three participants (25%) reported that they sometimes had problems categorizing the type of contact (both setting and relationship context): For example, when contact took place “on the street,” or when it was difficult to decide the relationship context—that is, a service clerk who was also a friend. Although it was possible to enter such contact information as a user-defined text, it would have required extra time, which was perceived as being “less convenient.”

As we previously indicated, the use of seek-bar widgets was associated with some difficulties. This was confirmed with responses stating that this type of control was “difficult to use,” “too sensitive to be exact,” “took time to move,” or as one participant put it, felt they had the “wrong fingers for sliders.” Difficulties moving the location marker on the map were also reported by two participants (17%). One participant (9%) noted that the default selection made them believe that the measure was preset to a midpoint value and that after realizing this was not the case, they found it difficult to move back to a midpoint value. Other problems included a technical problem that resulted in not being able to decline the reporting of a measure. Finally, for one participant (9%), it was not intuitive how to exit the number pad when entering a contact’s age, and another participant found that the predefined values for the duration slider were not as required (i.e., it was not possible to choose a value between 1 and 5 min).

#### Information provision

The overall ease of information provision was in many cases well above the mid-point of the scale (see Table 4). The most difficult app measures for participants to provide information on were outgroup typicality (*M* = 3.75, *SD* = 1.22) and relative status (*M* = 3.75, *SD* = 0.87), closely followed by contact duration (*M* = 3.83, *SD* = 0.94) and outgroup attitude (*M* = 3.91, *SD* = 1.04). No significant correlations were found between the ease of information provision per app measure and the number of contacts.

##### Differences in conceptual interpretation of measures

The qualitative analysis revealed that especially the concept of outgroup typicality was demanding. For example, one participant found that “It was so hard to decide [. . .] some old people seem old if we focus on the physical side, but in terms of psychological issues they seem very young,” whereas another participant felt that “group typicality is multidimensional and situational,” and a further participant found the term *typicality* simply “difficult to understand.” On occasions in which a contact was repeated with the same person, one participant tried to remember the previous rating, but then stated that they realized that outgroup typicality and status are “context-dependent.” Status was also reported as sometimes being difficult to judge. For example, one participant reported “I wasn’t sure because you really don’t know the people [that] well,” and another found that it was difficult because of “cultural reasons.”

#### Confidentiality, privacy, and covert usage

Although participants were informed that if they had any confidentiality or privacy concerns in regard to the contact partner, the contact situation or themselves, they could choose not to log a particular contact. Some minor issues arose on occasion about taking photos and logging the contact in near time.

##### Taking photos

Although it was hoped that such additional data would provide further novel insights into where and in which situations intergroup contact took place, the photo function was rarely used. One participant reported that they had taken photos but deleted them, as they “inadvertently included people/strangers in the background.” Two participants mistakenly thought that taking photos of the contact situation had been a requirement of the study but had forgotten to use the function, suggesting that the app could include a function that would prompt them. Finally, one participant noted that taking photos felt “too awkward, even when the person had left the contact situation.”

##### Logging contact

On rare occasions, logging contact without the contact partner noticing and while still in the vicinity of the contact location posed slight difficulties. As one participant reported, “the contact went on for [so] long because they were sat opposite, so even though the contact had ended, they were still there.” Another participant noted that they “had to find a quiet spot after [the] contact to log [it].” Another participant reported “slight confidentiality concerns” regarding logging contact at work, as they worked with people, including older people, in a therapeutic setting.

## Discussion

The purpose of this study was to test the usability and suitability of the Contact Logger—a smartphone application that allows the repeated assessment of contact experiences in near time and in a real-world environment. The results of this study demonstrate that the app is a viable and user-friendly tool that can help to collect data on interpersonal and intergroup encounters. In addition to self-reports, it can record location data and photos. Although this approach is not new to social psychology or behavioral research in general (e.g., Monk, Heim, Qureshi, & Price, 2015; Newton-Fisher, 2012), it is, to the best of our knowledge, a new approach within the field of intergroup contact research.

In the following section, we first discuss feedback from our sample on the usability of the app. Second, we will evaluate advantages and potential limitations of the app for intergroup contact research. Third, we will briefly suggest areas of future research in which the Contact Logger might be usefully employed.

### Usability

**Fig. 6:**
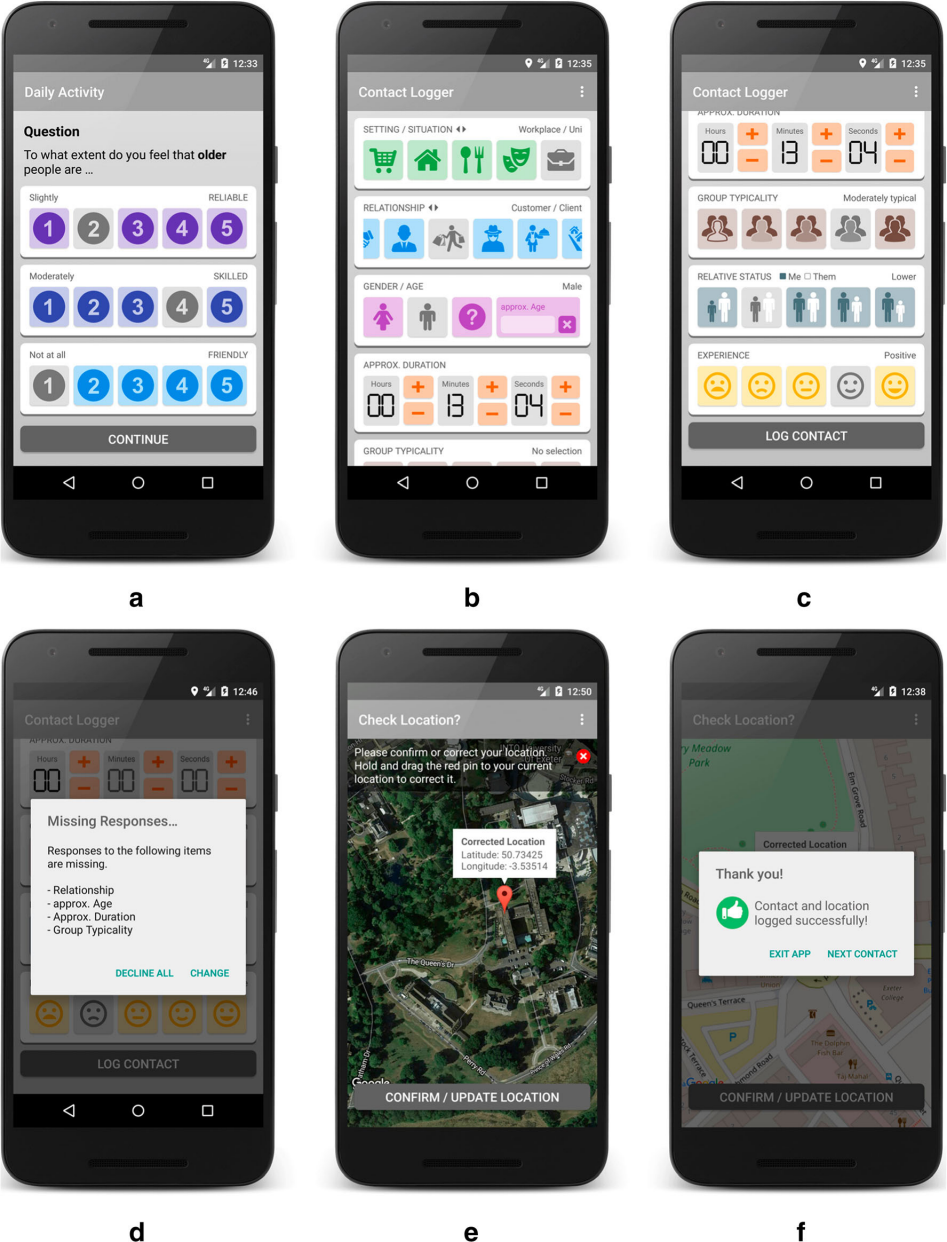
Screenshots of Contact Logger application (Version 2): (a) daily activity, shown once a day at start; (b) contact details, (c) contact details continued, scroll view; (d) dialog about missing contact attributes; (e) current location; (f) confirmation of successful logging of a contact.

Results of the usability study showed that the app was easy to use, with participants reporting only minor issues. The main issues related to the unsatisfactory sliding functionality of the seek-bar widgets, the timing of the daily attitude measure, and the optional photo feature. Except for the optional photo function, the nonsignificant correlations between the number of contacts logged with ‘ease of app use’ and ‘ease of information provision’ showed that these issues did not influence user behavior unduly. The outcomes of the usability study led to recommendations aimed to further refine the implementation of the Contact Logger for future research. The suggested modifications, listed in Table 5, address areas and specific functions for which participants experienced problems or found the interface/information unclear. The listed changes were implemented in Version 2 of the Contact Logger (see Fig. 6).

**Table 5:** Contact Logger: Proposed changes (Version 1) and implementation in Version 2

#	Issues identified in Version 1	Version 2
1	**Replacement for seek-bar widgets** Thumb element for measures group typicality, relative status and duration was insensitive and difficult to move. Problem confirmed by multiple users. Change design.	Resolved
2	**Improve duration measure** Setting of exact duration was not possible—that is, only in 24 predefined steps. Redesign duration measure to allow seamless duration entry.	Resolved
3	**Display and measurement of daily attitude** If the first contact of the day occurred after 4:00 p.m., the attitude screen was not shown, causing major data loss of the primary outcome measure. Also, consider changing to a multi-item measure to improve reliability.	Resolved / changed to three-item measure
4	**Improve action feedback** After logging of a contact, success of action was not clear enough. Clear notification is needed requiring confirmation—for example, give users the choice to continue or exit the app.	Resolved
5	**Add additional contact types** Participants reported difficulties in assigning certain types of contact to predefined categories. One or two additional contact settings and relationships types are required.	Resolved via Instructions
6	**Reconsider photo option** This function was barely used by participants due to confidentiality and privacy issues. Also, option deemed as “awkward” by some participants. Consider removing/deactivating the function.	Deactivated due to GDPR issues and participant feedback
7	**Missing responses notification** Missing response notification did not behave correctly under certain conditions. Fix bug.	Resolved
8	**Improve offline map handling** Offline map can only be downloaded from an external server in app admin-area. This is time-consuming, as the map file-size can be very large. Add function so that it can be added/deleted from internal SD-card of the phone.	Resolved
9	**Provide better usage information** Some participants were unsure of whether retrospective logging of a contact was acceptable. Consider providing more detailed information (video) about what to do in various contact scenarios.	Resolved

As we previously mentioned, both status and typicality were measured using seek-bar widgets. However, some participants found these difficult to adjust, and participants might therefore not always have captured the desired value. As can be seen in Figs. 6a, 6b, and 6c, in Version 2 of the app, the seek-bar widgets were replaced with buttons, resolving the issue in a simple and effective manner.

In addition, the app logic was changed so that the daily attitude measure (see Fig. 6a) is now shown each day when the Contact Logger is first started. This change, together with the instruction that the app should be opened once a day (irrespective of whether a contact was made), ensures that attitude is assessed consistently on a daily basis. Finally, to aid later assessment, the response date and time for the daily attitude measure are now recorded separately. These changes, along with the others listed in Table 5, aim to improve the data quality and the overall usability of the Contact Logger.

### Availability

The revised version of the Contact Logger (i.e., Version 2, which includes the changes and recommendations mentioned in Table 5) is open-source. Both the source code and a precompiled, ready-to-install Android package (APK), can be downloaded via https://www.contactlogger.app. Further development and modularization of the app^12^ is planned: This would allow different types of measures and icons to be chosen via an online or integrated plugin configuration menu, enabling the measurement of specific variables of interest or expanding its use to other research areas. Moreover, to make the Contact Logger more widely usable, a native iOS is planned. Finally, researchers with specific requirements can contact the authors to discuss the costs for the development of a custom version.

### Suitability

In addition to good usability, the app also appears well-suited for intergroup contact research: The number of contacts logged during the duration of the study was promising, even though the sample included mostly students and university staff, whose daily routine and environment may not offer as much contact with older people as other groups may have. The ability to record single contact events in near time, rather than relying on retrospective aggregated estimates of contact, promises greater ecological validity and an opportunity to study the dynamics and locatedness of intergroup contact further.

However, the extent to which the use of the app or the participation in the usability study encouraged participants to seek more contact than they would otherwise have had remains unclear. For example, it is plausible that the act of logging contact events over the course of several days or weeks may be perceived as game-like, nurturing collection instincts, despite the lack of a competitive environment (McIntosh & Schmeichel, 2004; Sobel, 2008; see also van Berkel, Goncalves, Hosio, & Kostakos, 2017, for possible benefits of mobile ESM gamification). This also raises the question of the extent to which the use of the app may function as a kind of intervention tool. Future studies should investigate this aspect more closely.

Furthermore, asking participants to respond on a frequent basis using a technical device that needs to be accessible at all times can be demanding and time-consuming. In some cases, this may lead to compliance problems, such as retrospective logging of contact events, especially on occasions when participants have forgotten to carry the device with them. Future studies should consider allowing users to install the app on their personal phones. This would increase convenience and engagement with the app (e.g., participants would not need to carry two phones). It would also reduce the administrative effort required on the part of the researcher: Participants would not need to collect nor return a phone, which also reduces the risk of phones not being returned or being damaged. Further development could expand usage to phones with different operating systems (e.g., iOS or Linux-based OSs). Although Gartner (2018) report that in the current global smartphone market, 85.9% of phones run Android OS, the popularity and competitive market share of Apple devices, running iOS, should not be ignored, especially in more affluent countries, where the majority of social psychology research is typically carried out. Nevertheless, in this particular study, we believe that it was justified to use the same make and model of mobile phone in order to aid measurement precision (e.g., GPS accuracy), to provide a robust user experience, and to ensure a standardized testing environment.

To minimize the time it took to log a contact, single-item measures from previous intergroup contact research were used (see Barlow et al., 2012; Hewstone, Cairns, Voci, Hamberger, & Niens, 2006; Pettigrew, Christ, Wagner, & Stellmacher, 2007; Stefaniak & Bilewicz, 2016). The key difference between the single-item measures used in the Contact Logger and single-item measures in self-reports is that the Contact Logger captures single contact events in near time, which can then be aggregated to a reliable index across several contact events in line with the research question (e.g., contact per day/per week/during an event). Furthermore, single-item measures of attitudes have shown good reliability (Dasgupta & Greenwald, 2001; Haddock et al., 1993; Tausch et al., 2010).

### Future directions

Researchers have suggested that intergroup contact needs to be studied in a way that recognizes intergroup dynamics as well as the situated nature of intergroup interactions (e.g., Dixon et al., 2019). In addition, recent studies have begun to examine fleeting interactions with strangers in intergroup contexts (e.g., Thomsen & Rafiqi, 2018).

The Contact Logger’s usability and functionality are highly optimized to support such research questions by allowing participants to quickly assess interactions in near time in their natural environment, while at the same time capturing the precise geographic location of contacts. Such research can help us to understand how geographical and architectural variables moderate the effect of contact on intergroup relations, thereby closing the interdisciplinary gap with diversity research conducted by human geographers (e.g., Wessel, 2009). Furthermore, the Contact Logger may also be useful for research that seeks to design effective interventions embedded in individuals’ everyday experiences, for instance through the optimization of public interaction spaces (see Bloomfield, 2013; Gustafson, 2001). Such interventions would fulfill the optimal contact criteria of voluntary and repeated interactions suggested by Pettigrew (1998).

We want to emphasize that the app is by no means limited to intergroup contact research. It may also prove useful for research on interpersonal encounters, including research on interactions with strangers (e.g., Epley & Schroeder, 2014), dating, children making friends, social support following bereavement, depression or a cancer diagnosis, and similar research questions.

Additionally, the app’s functionality could be expanded in several ways. For example, to gain a more in-depth insight into how the experiences of different types of contact in various contexts are perceived, conceptualized and interpreted, qualitative measures could be integrated. These could be text, audio or image-based. Previous studies that have already experimented with the collection of image-based data include a study about the experiences of new neighborhood contact in Belfast (Stevenson & Sagherian-Dickey, 2015) and a study exploring the experience and concerns related to the automatic capturing of everyday life through images (Price et al., 2017).

Technological advances and the continuous development of new software and sensors that provide high-quality location, proximity, visual, auditory data, and face and voice recognition systems are ongoing processes (see Benavides et al., 2011; Choudhury, 2004; Elrefaei, Alharthi, Alamoudi, Almutairi, & Al-Rammah, 2017; Niu, Wang, & Lu, 2015). Thus, future possibilities that could provide additional data include the integration of measures from external sensors: for example, sensors capable of measuring biological signals (e.g., heartbeat, skin-resistance, gait, etc.), or sensors that can detect the proximity and spatial distance of another person (Liu & Striegel, 2011).

## Conclusion

In sum, the Contact Logger is a new, easy-to-use tool that allows researchers to understand interpersonal and intergroup interactions in a more dynamic way, providing better ecological validity due to near-time measurement and additional information through geographic location logging and features such as photos. Being able to capture and measure the dynamic nature of contact in diverse neighborhoods and public spaces can help inform researchers and policymakers. It can help evaluate and make visible hotspots of intergroup conflict and tensions, as well as the effectiveness of interventions—for example, through the optimization of public interaction spaces (see Bloomfield, 2013; Gustafson, 2001).

## Footnotes

1 *Self-reports*, unless otherwise stated, refer here to retrospective online or paper-and-pencil surveys.

2 No information is available about the remaining 30%. Presumably, they cover experimental methods or intergroup contact research that does not focus on direct, face-to-face contact.

3 The terms EMA and ESM are often used interchangeably by researchers.

4 Technically challenging, as GPS typically works only outdoors, not indoors.

5 Android Studio, with Java as the programming language.

6 A secure online file host and system for tracking changes in computer files.

7 Tested extensively during the development phase.

8 https://osf.io/udc9a

9 Meaning the participant’s home.

10 https://developer.android.com/reference/android/location/Location.html#getAccuracy()

11 MAXQDA, Version 12

12 This will also apply to the free, open-source version, in that basic integration of newer features will be possible as the app is further developed.
